# RNAi-Mediated Knockdown of Chitin Synthase 1 (*CHS1*) Gene Causes Mortality and Decreased Longevity and Fecundity in *Aphis gossypii*

**DOI:** 10.3390/insects11010022

**Published:** 2019-12-26

**Authors:** Farman Ullah, Hina Gul, Xiu Wang, Qian Ding, Fazal Said, Xiwu Gao, Nicolas Desneux, Dunlun Song

**Affiliations:** 1 Department of Entomology, College of Plant Protection, China Agricultural University, Beijing 100193, China; farmanullah@cau.edu.cn (F.U.); gulhina680@gmail.com (H.G.); wangxiaoxiu1991@sina.com (X.W.); 15290882730@126.com (Q.D.); gaoxiwu@263.net.cn (X.G.); 2 Department of Agriculture, Abdul Wali Khan University, Mardan 23200, Khyber Pakhtunkhwa, Pakistan; dr.fazal@awkum.edu.pk; 3 Université Côte d’Azur, INRA, CNRS, UMR ISA, 06000 Nice, France; nicolas.desneux@inra.fr

**Keywords:** cotton-melon aphid, chitin synthase 1, RNA interference, mortality, longevity, fecundity

## Abstract

Chitin is a vital part of the insect exoskeleton and peritrophic membrane, synthesized by chitin synthase (CHS) enzymes. Chitin synthase 1 (CHS1) is a crucial enzyme in the final step of chitin biosynthetic pathway and consequently plays essential role towards insect growth and molting. RNA interference (RNAi) is an agent that could be used as an extremely target-specific and ecologically innocuous tactic to control different insect pests associated with economically important crops. The sole purpose of the current study is to use *CHS1* as the key target gene against the cotton-melon aphid, *Aphis gossypii*, via oral feeding on artificial diets mixed with dsRNA-*CHS1*. Results revealed that the expression level of *CHS1* gene significantly decreased after the oral delivery of dsRNA-*CHS1*. The knockdown of *CHS1* gene caused up to 43%, 47%, and 59% mortality in third-instar nymph after feeding of ds*CHS1* for 24, 48, and 72 h, respectively, as compared to the control. Consistent with this, significantly lower longevity (approximately 38%) and fecundity (approximately 48%) were also found in adult stage of cotton-melon aphids that were fed with ds*CHS1* for 72 h at nymphal stage. The qRT-PCR analysis of gene expression demonstrated that the increased mortality rates and lowered longevity and fecundity of *A. gossypii* were attributed to the downregulation of *CHS1* gene via oral-delivery-mediated RNAi. The results of current study confirm that *CHS1* could be an appropriate candidate target gene for the RNAi-based control of cotton-melon aphids.

## 1. Introduction

The cotton-melon aphid, *Aphis gossypii* Glover (Hemiptera: Aphididae) is a sucking and polyphagous insect pest infesting numerous species of plants worldwide, particularly Cucurbitaceae (melon, marrow, zucchini, watermelon) [1,2]. The direct damages of this pest are caused by curling and distorting the fresh leaves and twigs through direct feeding [3]. The indirect damages of *A. gossypii* are caused by transmitting more than 100 plant viruses, especially cucumber mosaic virus (CMV) [2], and by releasing a sugar-rich and sticky liquid material, i.e., honeydew, that also favors the development of fungus in the form of black sooty mold [4]. This pest act as a vector for transmitting 76 different viral diseases in more than 900 host plant species [5]. The application of chemical insecticides is considered crucial for the control of *A. gossypii* [6,7]. However, the frequent use of chemical insecticides leads towards resistance and hormesis effects resulting in significant control failures of insect pests [8,9,10], as well as potential multiple side effects on beneficial arthropods [11]. Resistance against synthetic chemical insecticides such as organophosphates, carbamates, pyrethroids, and neonicotinoids have been reported in *A. gossypii* throughout the world [12,13]. *Aphis gossypii* also showed resistance against imidacloprid, clothianidin, acetamiprid, thiacloprid, and thiamethoxam [13,14].

RNA interference (RNAi), a mechanism of post-transcriptional gene silencing that enables the downregulation of gene expression by artificial RNA molecules [15,16], was initially exposed in *Caenorhabtidis elegans* by Fire et al. [17]. In the last two decades, RNAi has been established as a molecular tool to silence key gene transcripts in many of insects from orders such as Coleoptera, Hemiptera, Diptera, Hymenoptera, and Lepidoptera [18,19,20,21,22]. Owing to its specificity, RNAi is also used as a new strategy for the control of a large number of pest species [23]. The selection of target gene and delivery method is crucial for the successful RNAi-based pest management [24]. The target gene for RNAi should be disastrous for the pest and harmless for the non-target insects and human beings [25]. The direct injection of dsRNA into insect body cavity and oral delivery techniques (such as droplet feeding and mixing with simulated diet) have been successfully used in several insect pests [21,26,27,28,29]. However, oral techniques minimize the side effects of microinjections and thus may be an efficacious method for RNAi-based control of *A. gossypii* [30,31].

Chitin is a major constituent of insect exoskeleton/cuticle and peritonea membranes, having a key part in the growth and molting process of insects [32,33]. The balance between the disintegration and formation of new chitin is essential for molting process and growth in insects, which are synchronized by different enzymes [34]. Chitin synthase (UDP-*N*-acetyl-d-glucosamine: chitin 4-β-*N*-acetylglucosamine transferase, EC 2.4.1.16) is a transmembrane protein, having major role in chitin synthesis [32,35]. Chitin synthase enzymes are encoded by two genes, i.e., Chitin synthase 1 (*CHS1*) and chitin synthase 2 (*CHS2*) [34]. *CHS1* is expressed in the epidermal cells encoding enzymes that are essential for the catalysis of chitin production in cuticle [36,37], while *CHS2* is expressed in the peritrophic membrane regulating enzymes for chitin production in insect midgut [38]. Several studies reported that *CHS1* gene is crucial for chitin synthesis in aphid molting [39,40,41]. However, due to lacking peritrophic membrane, *CHS2* was not existent in some insects [40,41,42]. Thus, for the suppression of chitin biosynthesis, *CHS1* is the key candidate gene for RNAi-based insect control.

In the current study, we used an artificial diet for the delivery of dsRNA to knockdown *CHS1* gene in cotton-melon aphid, *A. gossypii*. Moreover, the aphid mortality, longevity, and fecundity were investigated after feeding on dsRNA-*CHS1*. Results of the current study suggest that RNAi-mediated silencing of *CHS1* gene could be a promising novel bio-pesticide for the long-term control strategy against cotton-melon aphid.

## 2. Materials and Methods

### 2.1. Insect Culture

Developing cotton-melon aphid (*A. gossypii*) individuals were collected from Weifang District, Shandong Province, China. A colony of *A. gossypii* was established in the laboratory and was maintained on insecticide-free cucumber plants under standard laboratory conditions (25 ± 1 °C; 75% RH; 16:8 L:D) at China Agricultural University.

### 2.2. Total RNA Extraction and cDNA Synthesis

Total RNA was extracted from different instars (first, second, third, and fourth) of nymphs and adults of *A. gossypii* using TRIzol^®^ reagent (Invitrogen, Carlsbad, CA, USA), adopting the manufacturer’s instructions. Thirty surviving individuals from each group were pooled as one biological replicate. The RNA purity and concentrations were analyzed with a NAS-99 spectrophotometer (ACTGene). The cDNA was synthesized from 1 μg total RNA using the PrimeScript^®^ RT Reagent Kit with the gDNA Eraser (Takara, Dalian, China), following the instructions given by the manufacturer.

### 2.3. Preparation of Double-Stranded RNA (dsRNA)

*CHS1* dsRNA (498 bp) was synthesized by Transcript Aid T7 High Yield Transcription Kit (Thermo Fisher Scientific, Waltham, MA, USA) using cDNA obtained from third-instar nymphs following the manufacturer’s recommended procedure. The T7 RNA polymerase promoter sequence 5′-TAATACGACTCACTATAGGG-3′ was added in front of the forward and reverse primers as required for subsequent dsRNA synthesis (Table 1). PCR was performed at 94 °C for 3 min, followed by 35 cycles of 94 °C for 30 s, 56 °C for 30 s, and 72 °C for 30 s and an additional final polymerization step of 72 °C for 10 min. cDNA and single-stranded RNA were detached from the transcription reaction by DNase and RNase treatments. The dsRNA was purified by using phenol (pH 4.7) chloroform extraction and ethanol precipitation method and eluted in diethyl pyrocarbonate (DEPC)-treated nuclease-free water. A 714 bp fragment of green fluorescent protein (GFP) dsRNA as control was amplified using same conditions as discussed above with primers shown in Table 1. The dsRNA was quantified using a NAS-99 spectrophotometer (ACTGene, Piscataway, NJ, USA), and the integrity was analyzed by gel electrophoresis (1% agarose). DEPC water was used as the negative control.

### 2.4. Dietary Delivery of the Double-Stranded RNA (dsRNA)

The ds*CHS1* (dsRNA of *CHS1*), dsGFP (dsRNA of GFP), or DEPC water was mixed in the synthetic feeding diet (0.5 mol/L sterile sucrose solution) at an ultimate concentration of 100 ng/μL and fed to the third-instar nymphs. For the in vitro feeding assays, sterilized glass tubes (3 cm in length and 2 cm in diameter) that open at both ends were used [43,44]. One end of each glass tube was wrapped with two coatings of parafilm membrane that contained a mixture of synthetic diet sandwiched between the two layers of parafilm. Fifty apterous adult aphids were placed into the tube with a fine brush. The tube was impenetrable with a piece of Chinese art paper (Xuan paper). The glass tubes were kept under standard laboratory conditions (25 ± 1 °C; 75% RH; 16:8 L:D). Mortality of cotton-melon aphids was recorded at 12, 24, 48, and 72 h post-feeding. All the experiments were repeated three times.

### 2.5. Quantitative Real-Time PCR (RT-qPCR)

Quantitative real-time PCR (RT-qPCR) was accomplished on the Applied Biosystems 7500 Real-Time PCR system (Applied Biosystems, Foster, CA, USA) using SYBR^®^ Premix Ex Taq™ (Tli RNaseH Plus) (Takara, Dalian, China) to analyze the expression level of *CHS1* either from N1 to N4 and adults, as well as the nymphs after 12, 24, 48, and 72 h after treatment with RNAi. Primers for the *CHS1* were designed based on the conserved sequence of soybean aphid, *Aphis glycines* Matsumura (Hemiptera: Aphididae) *CH1* gene (GenBank Accession No. JQ246352.1). All primers were synthesized using PRIMER 3.0 (http://bioinfo.ut.ee/primer3-0.4.0/) (Table 1). The reaction of qRT-PCR was performed in a 20 μL volume of a mixture, which contained 10 μL of SYBR^®^ Premix Ex Taq, 7.8 μL ddH_2_O, 0.4 μL of ROX, 0.4 μL of each primer, and 1 μL of the cDNA. The qRT-PCR conditions were 95 °C for 30 s, followed by 40 cycles of 95 °C for 5 s and 60 °C for 34 s, and then one dissociation step cycle of 95 °C for 15 s, 60 °C for 1 min, 95 °C for 30 s, and 60 °C for 15 s. The qRT-PCR analysis was repeated in triplicate. The standard curve was established with serial dilutions of cDNA (1, 1/10, 1/100, 1/1000, 1/10,000, and 1/100,000) to check the amplification efficiencies and cycle threshold (Ct). Quantification of gene transcriptions was conducted using the 2^−∆∆Ct^ method [45]. *EF1α* (GenBank Accession No. EU019874.1) and *β-ACT* (GenBank Accession No. KF018928.1) were used as the internal control [46].

### 2.6. Longevity and Fecundity Analysis

Newly emerged apterous adults were collected after constant feeding of dsRNA for 72 h at third instar nymphal stage. Each aphid was shifted to a fresh cucumber seedling. Thirty individuals per experimental group (DEPC-water, dsGFP, and dsCHS1) were used and each aphid was considered as a single replicate [6,8]. The longevity and fecundity (number of newly-born nymphs/individual aphid) were recorded daily until the aphids died. The neonate nymphs were detached from the seedlings after counting. Insecticide-free cucumber seedlings were replaced on a weekly basis throughout the experiment.

### 2.7. Data Analysis

The data related to longevity, fecundity, and gene expression of *A. gossypii* were statistically analyzed using one-way analysis of variance (ANOVA) with Tukey’s post hoc test (IBM, SPSS Statistics, version 22). *p* < 0.05 was supposed to be significant for all tests.

## 3. Results

### 3.1. Expression of CHS1 Gene among Growth Stages of A. gossypii

**Figure 1:**
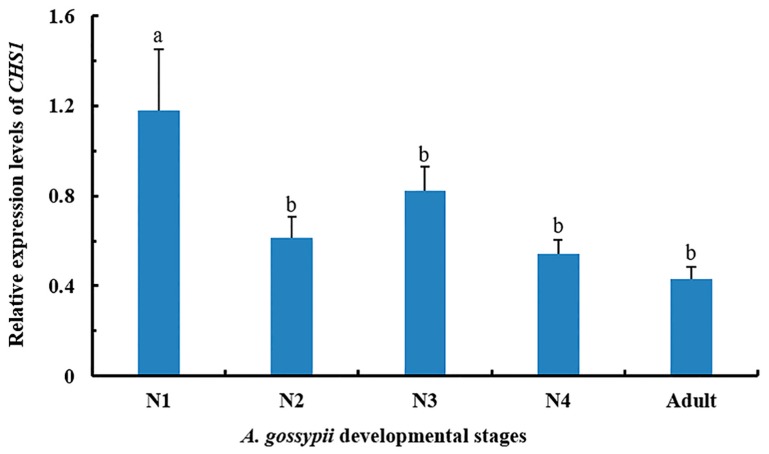
Relative expression pattern of *CHS1* gene at different developmental stages of *A. gossypii* analyzed by qRT-PCR. The expression level is expressed as the mean (±SE) of the three biological replicates, and thirty insects were used per pooled RNA sample. Letters above the bars represent significant differences at *p* < 0.05 level (one-way ANOVA followed by Tukey’s HSD test). *EF1α* and *β-actin* are used as the internal control. N1: first instar; N2: second instar; N3: third instar; N4: fourth instar.

The relative expression level of *CHS1* gene among different growing stages of *A. gossypii* was assessed by qRT-PCR. The mRNA transcriptions were detected in all life stages. However, the expression of *CHS1* gene was highest during the first instar of nymphal stage compared to the second, third or fourth instar as well as adult growth stage (Figure 1). No significant differences were observed in the expression level of *CHS1* gene in the second instar, third instar, fourth instar, and adult stage aphids.

### 3.2. Functional Analysis of CHS1 by RNAi

**Figure 2:**
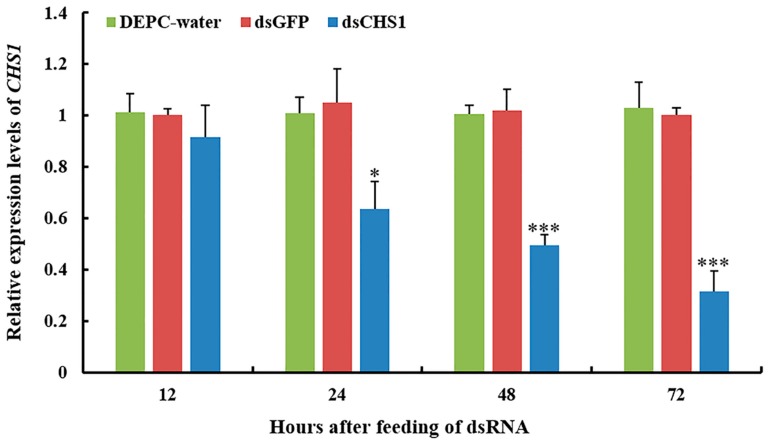
Gene silencing of *CHS1* in *A. gossypii* fed an artificial diet with or without dsRNA-*CHS1*. Relative abundance of *CHS1* gene transcripts were determined as mean (±SE) of the three biological replicates, and thirty insects were used per pooled RNA sample with control as the calibrator, i.e., cDNA from non-RNAi aphids (only fed on artificial diet with DEPC-water and dsGFP). *EF1α* and *β-actin* are used as the internal control. Treatments were compared using one-way ANOVA (Tukey’s HSD test, *p* < 0.05). * and *** represent *p* < 0.05 and *p* < 0.001, respectively.

Quantitative real-time PCR (RT-qPCR) was used to scrutinize the silencing efficiency of ds*CHS1* in aphids. We investigated the expression level of the *CHS1* gene after continuous oral delivery of dsRNA to the third-instar nymph of *A. gossypii* for 12, 24, 48, and 72 h (Figure 2). RT-qPCR analysis indicated significantly (*p* < 0.05) reduced transcript level of *CHS1* mRNA after the oral uptake of dsRNA-*CHS1* for 24 h, as compared with the DEPC-water and dsGFP control treatments. After 48 and 72 h of ingestion, dsRNA-*CHS1* caused very significant (*p* < 0.001) reduction in *CHS1* mRNA abundance, indicating a substantial gene silencing (Figure 2). However, no significant differences were observed for *CHS1* mRNA transcript level at 12 h post-feeding of dsRNA-*CHS1*, as compared with positive and negative control treatments.

### 3.3. Effects of dsRNA-CHS1 on Aphid Mortality, Longevity, and Fecundity

**Figure 3:**
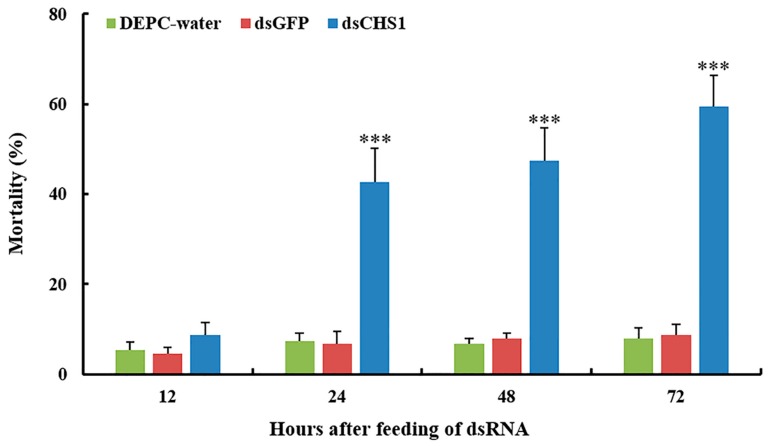
Mortality rates (%) of *A. gossypii* fed with artificial diet containing DEPC-water, dsGFP, and ds*CHS1* at third instar nymphal stage over time. The values are presented as the mean (±SE) of three replications (50 insects were used per replicate). Treatments were compared using one-way ANOVA (Tukey’s HSD test, *p* < 0.05). *** represents *p* < 0.001.

**Figure 4:**
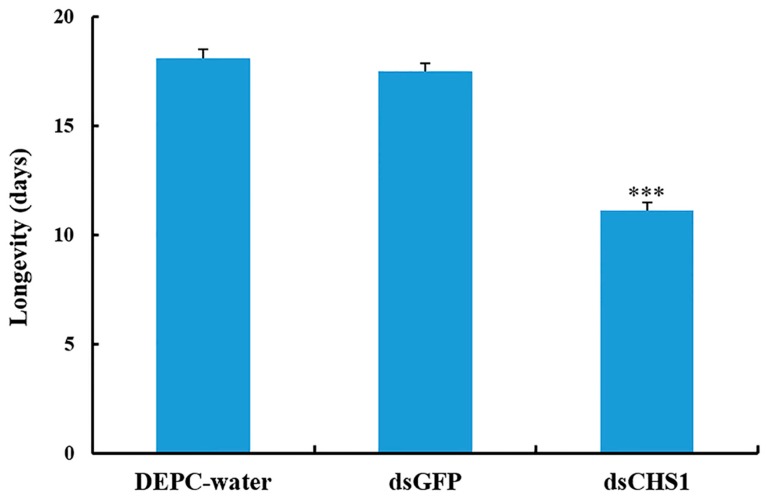
Impact of *CHS1* gene silencing on adult longevity of *A. gossypii*, previously fed artificial diets containing DEPC-water, dsGFP, and ds*CHS1* at their nymphal stage (third instar). The values are presented as the mean (±SE). Thirty individuals per experimental group (DEPC-water, dsGFP, and dsCHS1) were used, and each aphid was considered as a single replicate. Treatments were compared using one-way ANOVA (Tukey’s HSD test, *p* < 0.05). *** represents *p* < 0.001.

**Figure 5:**
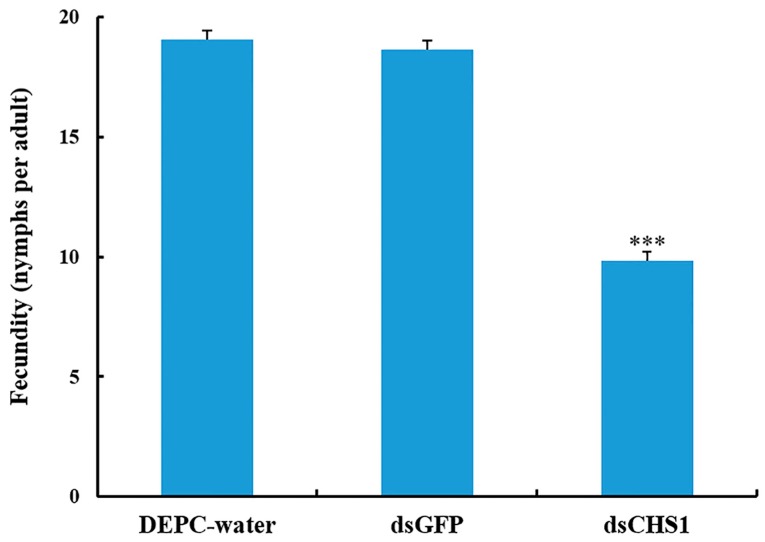
Effect of *CHS1* gene silencing on fecundity of *A. gossypii*, previously fed artificial diets containing DEPC-water, dsGFP, and ds*CHS1* at their nymphal stage (third instar). The values are presented as the mean (±SE). Thirty individuals per experimental group (DEPC-water, dsGFP, and dsCHS1) were used and each aphid was considered as a single replicate. Treatments were compared using one-way ANOVA (Tukey’s HSD test, *p* < 0.05). *** represents *p* < 0.001.

No considerable aphid mortality was seen after feeding of ds*CHS1*, dsGFP, and DEPC-water for 12 h. However, a significantly increased mortality (*p* < 0.005) of *A. gossypii* was observed at 24, 48, and 72 h post-feeding of dsRNA-*CHS1*, as compared with the positive and negative control treatments (Figure 3). The average mortality of *A. gossypii* reached 43%, 47%, and 59% after continuous feeding of ds*CHS1* for 24, 48, and 72 h, respectively. The mortality of aphids treated with DEPC-water at 24, 48, and 72 h was 7%, 7%, and 8%, respectively, while the mortality for aphids fed on dsGFP was 7%, 8%, and 9% at 24, 48, and 72 h post-feeding, respectively (Figure 3). The continuous feeding of ds*CHS1* for 72 h significantly (*p* < 0.005) reduced the longevity of *A. gossypii* (approximately 38%), compared with the dsGFP and DEPC-water treated groups (Figure 4). Similarly, a significant (*p* < 0.005) decline was noticed in the fecundity of surviving *A. gossypii* (approximately 48%) prior to dietary delivery of ds*CHS1* for 72 h, as compared to control treatments (Figure 5).

## 4. Discussion

Chitin synthase 1 (*CHS1*) contributes significantly towards the formation of chitin in insects [32] and is extremely critical for insect molting [34,47,48,49]. Chitin synthase genes have been deliberated in a number of insect pest species, such as *A. gossypii*, soybean aphid (*A. glycines*), and brown citrus aphid (*Toxoptera citricida*) [7,40,41]. As chitin is absent in plants and mammals [47,48,50], RNAi-mediated silencing of genes related to the chitin synthesis pathways is a striking target for insect pest control. The abundance of *CHS1* mRNA transcript in migratory locust (*L. migratoria*) was at maximum in the adult stage and was lowest in the developmental stages [51]. However, in soybean aphid (*A. glycines* Matsumura), the mRNA transcription of *CHS1* gene was highest in the nymphal stage (second instar) [41]. Similarly, the peak expression level of *CHS1* was also detected during developmental stages of grain aphid (*Sitobion avenae*) [39]. In the present study, transcripts of *CHS1* gene were perceived in all life stages of *A. gossypii*; however, the highest expression was observed in the first-instar nymph. These results suggested that expression patterns of *CHS1* gene are different among different insect pests.

RNA interference is a gene-silencing technique that has been efficiently used for the last two decades in order to study the resistance mechanisms, functional analysis of detoxification genes, and control of insect pests through oral dietary delivery or artificial injection [26,52,53,54,55,56,57]. For the effective silencing of target genes, it is necessary to introduce the dsRNA molecule into the body of insect to disrupt the expression of target genes at the transcription level. RNAi-mediated gene knockdown has largely been adopted through oral dietary delivery, droplet-feeding, or injection of dsRNA [52,53,57,58,59]. RNAi-mediated insect control was initially studied by Mao et al. and Baum et al. [18,60]. They developed transgenic plants that express dsRNA for the control of cotton bollworm, *Helicoverpa armigera* (Lepidoptera), and Western corn rootworm, *Diabrotica virgifera* (Coleoptera). Afterward, many studies were conducted to target different insect genes through various methods, e.g., feeding, injection, soaking, and transgenic plants, which are summarized in certain previous works, such as those of Kola et al. and Kim et al. [25,61]. The difficulties and endorsements for the RNAi-mediated positive insect control were discussed in detail by Scott et al. and Burand and Hunter in their review papers [22,24]. 

Zhang et al. demonstrated that RNAi-mediated silencing of *CHS1* through injection with 1 μg μL^−1^ Lm*CHS1*, Lm*CHS1A*, and Lm*CHS1B* dsRNA causes 95%, 88%, and 51% mortalities in the oriental migratory locust, respectively [51]. Mohammed et al. reported approximately 30%, 55%, and 75% larval mortalities in the potato tuber moth (*Phthorimaea operculella*) after injection with 50, 100, and 200 ng 5′-dsRNA-*CHS1*, respectively [27]. Souza-Ferreira et al. showed a 25% decrease in transcripts of *CHS* after injection of dsRNA-*CHSe* in *Rhodnius prolixus* [37]. Moreover, the chitin deposition and eclosion were also affected in the first-instar nymph fed with dsRNA. Zhao et al. reported that plant-mediated RNAi of *Sitobion avenae CHS1* gene causes ~50% decreased transcription, whereas ~20% reduction was observed in number of grain aphid and ecdysis [39]. Our results showed that RNA-interference-mediated knockdown of the *CHS1* gene causes a significant (*p* < 0.001) reduction in the mRNA transcription after oral delivery of dsRNA-*CHS1* for 48 and 72 h. Moreover, 59% mortality of cotton-melon aphid was observed at 72 h post-feeding of ds*CHS1*. The mortalities of aphids treated with DEPC-water and dsGFP were 8% and 9% at 72 h post-feeding, respectively, which could be due to the side effects of controls, as reported previously [31,62]. In the present study, knockdown of *CHS1* gene also significantly (*p* < 0.005) reduced the longevity (approximately 38%) and the fecundity (approximately 48%) of the surviving cotton-melon aphids after oral uptake of dsRNA-*CHS1* for 72 h. These results demonstrated that the oral dietary delivery dsRNA-*CHS1* could lead to the decreased transcription level of *CHS1* and subsequently affect aphid development.

## 5. Conclusions

The results of this study revealed that knockdown of the chitin synthase 1 (*CHS1*) gene via oral delivery of dsRNA-*CHS1* in *A. gossypii* caused mortality. Moreover, oral-delivery-mediated RNAi of *CHS1* also disrupted the adult longevity and fecundity of the cotton-melon aphid. Furthermore, the current study proposes that *CHS1* might be a possible candidate gene to target with RNA interference technology. For the long-term control tactics of pests, this strategy might be more suitable for innovative management of *A. gossypii* in field conditions.

## Figures and Tables

**Table 1:** The specific primers used in dsRNA synthesis and qRT-PCR.

Primer Name	Primer Sequences
ds*CHS1*-F	**TAATACGACTCACTATAGGG**ACCATTTTAGGACCGGGAAC
ds*CHS1*-R	**TAATACGACTCACTATAGGG**CTTTTCTGACTCCTCAGCGG
dsGFP-F	**TAATACGACTCACTATAGGG**TGACCACCCTGACCTAC
dsGFP-R	**TAATACGACTCACTATAGGG**TTGATGCCGTTCTTCTGC
*CHS1*-F	ATTGCGTCACGATGATCCTT
*CHS1*-R	TGGTCGCTAGACGTTCACAC
*EF1α*-F	GAAGCCTGGTATGGTTGTCGT
*EF1α*-R	GGGTGGGTTGTTCTTTGTG
*β-Actin*-F	GGGAGTCATGGTTGGTATGG
*β-Actin*-R	TCCATATCGTCCCAGTTGGT

The bold capital letter shows the T7 RNA polymerase promoter sequence.
